# A randomized, double-blind, placebo-controlled trial of paracetamol and ketoprofren lysine salt for pain control in children with pharyngotonsillitis cared by family pediatricians

**DOI:** 10.1186/1824-7288-37-48

**Published:** 2011-09-29

**Authors:** Nicolino Ruperto, Luigi Carozzino, Roberto Jamone, Federico Freschi, Gianfranco Picollo, Marcella Zera, Ornella Della Casa Alberighi, Enrica Salvatori, Alessandra Del Vecchio, Paolo Dionisio, Alberto Martini

**Affiliations:** 1 IRCCS G Gaslini, Pediatria II, Reumatologia, PRINTO, Genoa, Italy; 2 Local Health Unit (Azienda Sanitaria Locale -ASL) 3 "Genovese", Genoa, Italy; 3 IRCCS G Gaslini, Scientific Direction, Clinical Pharmacology and Pediatric Clinical Trials Office, Genoa, Italy; 4 Headquarters Medical Department, ACRAF S.p.A, Rome, Italy; 5 IRCCS G. Gaslini, Pediatria II, Reumatologia and Dipartimento di Pediatria, Università degli Studi di Genova, Genoa, Italy

**Keywords:** paracetamol, ketoprofen lysine salt, placebo, randomized double blind clinical trial, family pediatricians

## Abstract

**Background:**

To evaluate the analgesic effect and tolerability of paracetamol syrup compared to placebo and ketoprofen lysine salt in children with pharyngotonsillitis cared by family pediatricians.

**Methods:**

A double-blind, randomized, placebo-controlled trial of a 12 mg/kg single dose of paracetamol paralleled by open-label ketoprofren lysine salt sachet 40 mg. Six to 12 years old children with diagnosis of pharyngo-tonsillitis and a Children's Sore Throat Pain (CSTP) Thermometer score > 120 mm were enrolled. Primary endpoint was the Sum of Pain Intensity Differences (SPID) of the CSTP Intensity scale by the child.

**Results:**

97 children were equally randomized to paracetamol, placebo or ketoprofen. Paracetamol was significantly more effective than placebo in the SPID of children and parents (*P *< 0.05) but not in the SPID reported by investigators, 1 hour after drug administration. Global evaluation of efficacy showed a statistically significant advantage of paracetamol over placebo after 1 hour either for children, parents or investigators. Patients treated in open fashion with ketoprofen lysine salt, showed similar improvement in pain over time. All treatments were well-tolerated.

**Conclusions:**

A single oral dose of paracetamol or ketoprofen lysine salt are safe and effective analgesic treatments for children with sore throat in daily pediatric ambulatory care.

## Background

Treatment of acute pain, particularly in pediatric population, should be a priority for clinicians. In the past, pain has been underestimated and sometimes undertreated in children, probably due to individual and social attitudes toward pain and the complexity of its assessment in children [1-3]. Nowadays, the importance of pain control in the pediatric population is widely recognized. However, there is still a lack of adequate clinical trials assessing the pharmacological effects of the oral analgesics commonly used in pediatric daily primary care [3,4].

Paracetamol (acetaminophen) is currently one of the most popular and widely used analgesic and antipyretic in children for the symptomatic treatment of acute pain and fever. Differently from non-steroidal anti-inflammatory drugs (NSAIDs), paracetamol does not produce gastrointestinal damage or untoward cardio-renal effects. On the other hand, its anti-inflammatory activity is negligible [5].

Aim of this trial was to evaluate and confirm the analgesic effect and the tolerability of a paracetamol syrup formulation administered at the dosage of 12 mg/kg in children with pharyngotonsillitis. The study was carried out in double-blind conditions in comparison to placebo and controlled, in an open fashion, with ketoprofen lysine salt 40 mg as the positive control.

## Methods

### Study design

The study design was a randomized, double-blind, parallel group, placebo-controlled trial of 12 mg/kg single dose paracetamol syrup with an open label comparison with ketoprofen lysine salt sachet 40 mg as the positive control over three days.

### Study setting and population

Between March 2006 and May 2007, the study was set in five pediatric primary care public ambulatories of the Italian national net of family pediatricians. The protocol was approved by the Ethics Committee and parents and children signed the approved informed consent form as appropriate.

Those eligible were 6-12 year-old with diagnosis of pharyngo-tonsillitis confirmed by a score > 5 in the Tonsillo-Pharyngitis Scale (TPS) [6], a score > 120 mm in the Children's Sore Throat Pain (CSTP) Thermometer [6], and a maximum 1-week disease duration. Subjects were excluded from the study if they had positive history of hypersensitivity or allergy to the study medications, other conditions know to interfere with assigned drugs, or if they used any antipyretic drugs or throat lozenges in the past 6 hours, and/or analgesics or any "cold" medication in the past 8 hours.

### Study treatments

**Figure 1:**
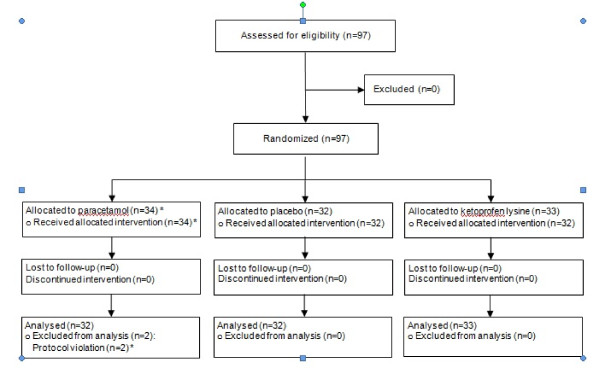
**Flow diagram of the progress of enrolled patients through the trial**.

After consent and baseline assessments, children were randomly assigned to 1 of the 3 groups (Figure 1). The first and second groups received a single administration of paracetamol syrup 12 mg/kg (Tachipirina^® ^syrup 2.4%, ACRAF SpA), corresponding to 1 mL/2 kg of body weight or placebo syrup (1 mL/2 kg of body weight) in a double blind fashion. The third group was assigned, to open label to ketoprofen lysine salt 40 mg (Oki^® ^80 mg granules for oral suspension, half sachet, Dompè SpA) in order to have an indirect comparison with a widely used analgesic in Italy. A double dummy design was not forecast for technical and logistical reasons. No further dosing was allowed in the following 4 hours. After the clinical assessments by the family pediatrician, patients assigned in a double blind fashion to the syrup (active or placebo) received a bottle of paracetamol for the home management of pain. The paracetamol dosage to be used was 12 mg/kg up to 4-5 times daily, and ketoprofen 40 mg (half 80 mg sachet) every 8 hours for a maximum of 3 administrations daily. The paracetamol 12 mg/kg single dose was chosen on the basis of the therapeutic range of 10-15 mg/kg widely recognized as effective in the treatment of fever and pain control [7] while the ketoprofen lysine salt dosage was the same as recommended in its own Summary of Product Characteristics.

There was one randomization sequence computer generated via Microsoft Access 2000. A pre-assigned list with progressive numbering was provided to each investigator. The double-blind conditions were obtained using paracetamol matching placebo syrup, including matching external box and internal opaque bottle. Ketoprofen lysine salt was not masked due to manufacturing issues, and was provided in sachets as per the current Italian marketing authorization.

### Outcome measures

Three assessments of pain intensity were performed at the primary care facilities (baseline, 30 minutes and 1 hour after treatment), and three at home (2, 3 and 4 hours after treatment). On day 4 children came back to their family pediatrician office for final visit assessment.

At baseline, the investigators assessed the patient's medical history, physical examination, concomitant treatments, and underwent a rapid antigen detection testing (Testpack Plus Strep-A OBC II^®^, Abbott). In case of positive findings on the test, an appropriate antibiotic for the treatment of streptococcal infection was prescribed. The pediatrician evaluated the severity of tonsillo-pharyngitis by the TPS a 0-3 categorical scale (score range 0-21) considering 7 clinical parameters: body temperature, tonsils' volume, pharyngeal' colour, enanthema, size, number and sensibility of anterior cervical lymph node. Children completed the CSTP thermometer as proposed by Schachtel et al [6], a vertical paper drawn 0-200 mm thermometer with anchoring words no pain (0 mm) and very severe pain (200 mm), divided at 10 mm intervals; the child was asked to swallow and to "color in the pain thermometer so that it shows how much your throat hurts now" At all fixed times, the child was also asked to indicate pain intensity using the horizontal five-faces of the Children's Sore Throat Relief Scale (CSTRS) in the version proposed by Schachtel et al [6]; this scale consisted of a series of five faces, from "no relief" on the left to "complete relief" on the right.

Before assigning the treatment, pain intensity was also independently rated by the investigator and parent (usually the mother) using the Sore Throat Pain Intensity (STPI), a 0-100 mm VAS, with anchoring words no pain (child has no difficulty in deglutition at 0 mm) and very severe pain (child has a lot of difficulties in deglutition at 100 mm) [6].

Both the investigator and parent evaluated pain intensity at 30 min and 1 hour after dosing, by the STPI Scale, while the child used the CSTP Intensity Scale. Using the same scale, the child and the parent also assessed at home pain intensity at 2, 3, and 4 hours after treatment. At day 4 after treatment period, a final visit was performed by the family pediatrician in its office to re-evaluate the patient's clinical conditions.

Efficacy was also evaluated by parent and investigator on a 5 levels categorical scale (very good, good, fair, poor or very poor) and by children on a 3 levels categorical scale (a lot, little or none).

In addition 1 hour after treatment, and at the final visit, the investigator verified the occurrence of adverse events during the study period and judged tolerability using a 5-point scale (from very good to very poor). Tolerability was also assessed by the parent at 1 and 4 hours after treatment.

The use of the CSTP Thermometer, and of the CSTRS happy-sad faces for efficacy were explained by the family pediatrician to the child by using pre-printed color images for adequate training. Training was also offered to the parent for the use of the STPI and overall efficacy and tolerability categorical scales. Both the child and the parent completed their baseline pain assessment before randomization and drug assignment. All intervention including physical examination, additional medication assessment scale were prospectively documented on ad hoc designed 3 carbon copy paper case report forms monitored by the Pediatric Clinical Trial Office of the G. Gaslini hospital.

### Statistics

The study complied with the Consolidated Standards of Reporting Trials (CONSORT) statement and used the intention-to-treat population for analysis [8-10].

Ketprofen lysine salt was used in open conditions as the positive control. Thus, comparisons between the group treated in open label and double blinded groups were descriptive in nature and no formal statistical comparison were performed with ketoprofen lysine salt [11].

Descriptive statistics were reported in terms of means and standard deviation (SD) or with 95% confidence intervals (95% CI) for quantitative variables and in terms of absolute frequencies and percentages for qualitative variables.

The following efficacy parameters were evaluated: Pain Intensity Difference (PID) calculated at each time by subtracting the baseline (CSTP and STPI) pain intensity score from the actual pain intensity score, Sum of Pain Intensity Differences (SPID) and Total Pain Relief (TOTPAR) estimated as the Area Under the Curve (AUC).

The analysis of variance was used to evaluate SPID and TOTPAR comparing paracetamol to placebo. SPID of CSTP Intensity scale was the primary study endpoint. All efficacy evaluations were analyzed by the Cochran-Mantel-Haenszel test comparing paracetamol to placebo. All the tests were two sided and a p value < 0.05 was considered statistically significant.

The sample size calculation was based on the results reported in a study comparing ibuprofen suspension (10 mg/kg) and paracetamol (15 mg/kg) to placebo in children with sore throat [6]. Sixty patients (30 patients per group) were adequate to detect a difference between paracetamol and placebo of 59 in SPID of CSTP Intensity scale, assuming a standard deviation of 88.8, using a two group t-test with a 0.05 one-sided significance level, and a power higher than 80%. A group of 30 patients treated with ketoprofen lysine salt was included in the trial as active control.

## Results

### Patient Enrollment and Baseline characteristics

Ninety-seven Caucasian school children (55 males and 42 females) with pharyngotonsillitis were recruited. Thirty-two were assigned to paracetamol, 32 to placebo, and 33 to ketoprofen lysine salt (Figure 1). Two patients, initially randomized in the placebo and ketoprofen group, had a subsequent episode of pharyngotonsillitis, were both randomly re-allocated in the paracetamol group but were excluded from the analysis.

Table 1 shows the demographic characteristics and mean baseline (SD) scores assessing the severity of pharyngotonsillitis and pain..

**Table 1:** Children disposition and demographics at baseline.

	paracetamol (n = 32)	placebo (n = 32)	ketoprofen (n = 33)	total (n = 97)
Males n (%)	20 (62.5%)	17 (53.1%)	18 (54.5%)	55 (56.7%)
Positive Strep-test (%)	16 (50.0)	15 (46.9)	15 (45.4)	46 (47.4)
	mean (SD)	mean (SD)	mean (SD)	mean (SD)
Age (years)	8.6 (1.9)	8.1 (1.7)	8.3 (1.9)	8.3 (1.8)
Height (cm)	133.8 (11.3)	132.7 (11.4)	133.3 (12.4)	133.3 (11.6)
Weight (kg)	30.7 (8.5)	30.0 (8.0)	33.9 (13.1)	31.6 (10.2)
TPS (0-21 points)	10.9 (2.1)	11.4 (2.8)	10.6 (2.9)	11.0 (2.6)
Temperature (°C)	37.7 (0.7)	37.9 (0.9)	38.0 (0.8)	37.8 (0.8)
CSTPI (0-200 mm VAS)	157.4 (17.3)^a^	158.2 (20.2)	158.2 (17.1)^b^	157.9 (18.1)^c^
STPI-parents (0-100 mm VAS)	63.3 (9.9)	63.9 (13.9)	64.1 (10.2)	63.8 (11.3)
STPI-investigators (0-100 mm VAS)	67.3 (11.5)	68.9 (14.4)	69.7 (12.1)	68.6 (12.6)

CSTPI = Children's Sore Throat Pain Intensity; STPI = Sore Throat Pain Intensity; TPS = Tonsillo-Pharyngitis Score

a) n = 31; b) n = 32; c) n = 95

### Efficacy and safety evaluation over time of paracetamol versus placebo

**Figure 2:**
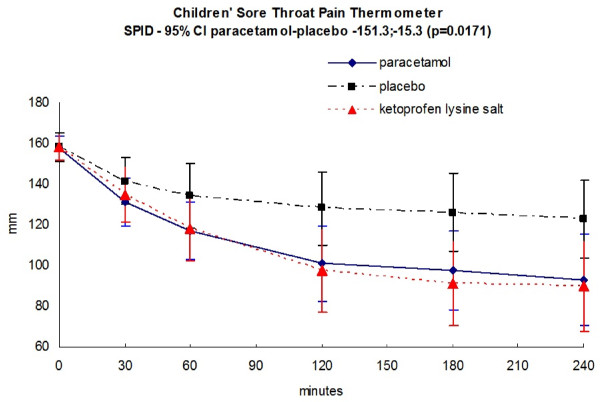
**Mean (95% CI) change over time course of pain as assessed by children with the Children's Sore Throat Pain (CSTP) Thermometer with values going from 0 mm (no pain) to 200 mm (very severe pain)**. P values refers to the as Sum of Pain Intensity Differences (SPID) with 95% CI for the comparison paracetamol versus placebo.

Figure 2 shows the results of primary outcome of the study, the time course of pain in each treatment group, as assessed by children with the CSTP. Paracetamol was significantly more effective than placebo in the SPID of children (two tailed 95%CI paracetamol-placebo, from -151.3 to -15.3, p = 0.0171). Similar results for the comparison paracetamol versus placebo were obtained when pain was assessed by children with of TOTPAR of the five-faces of the CSTRS (p = 0.0039).

**Figure 3:**
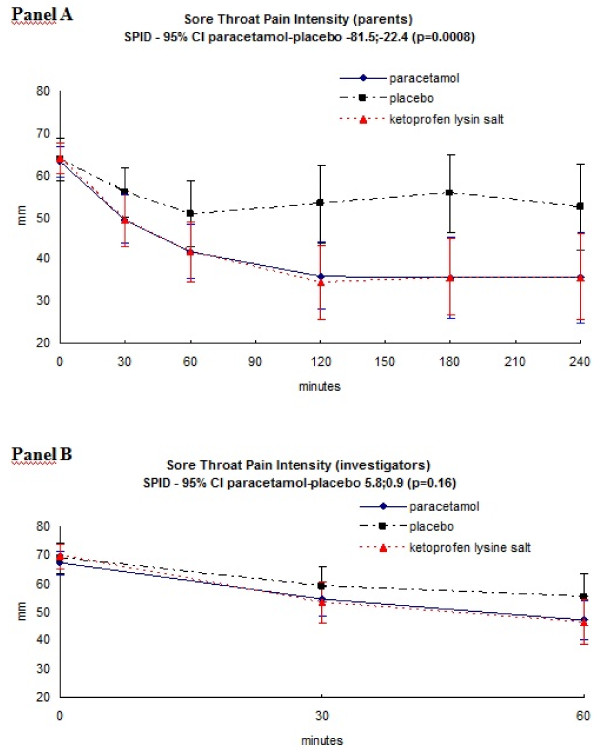
**Mean (95% CI) change over time course of pain as assessed by parents **(Panel A) **or investigators **(Panel B) **with the Sore Throat Pain Intensity (STPI) with values going from 0 mm (no difficulty in deglutition) to 100 mm (a lot of difficulties in deglutition)**. P values refers to the as Sum of Pain Intensity Differences (SPID) with 95% CI for the comparison paracetamol versus placebo.

Figure 3 shows the time course of pain in each treatment as reported by the SPID as measured by the STPI of parents (panel A) and investigators (panel B), respectively. Paracetamol was significantly more effective than placebo in the SPID of parents (p = 0.0008), while no differences between paracetamol and placebo were detected in the SPID reported by investigators.

Table 2 shows the overall categorical efficacy evaluation by children, parents and investigators at different time points. After 1 hour from dosing, a statistically significant advantages of paracetamol over placebo were detected in the judgement reported by children, parents and investigators. These results were confirmed in the at home assessment performed by children and parents, 4 hours from dosing and after 4-days.

**Table 2:** Efficacy evaluation by children (3 levels categorical scale: a lot, little or none), parent and investigator (5 levels categorical scale: very good, good, fair, poor or very poor).

	paracetamol (n = 32)	placebo (n = 32)	p values	ketoprofen (n = 33)
Children (a lot after 1 hour)	14 (44%)	7 (22%)	p 0.0156	18 (55%)
Children (a lot after 4 hours)	19 (59%)	5 (16%)	p 0.0003	18 (55%)
Parent (very good/good at 1 hour)	19 (59%)	8 (25%)	p 0.0016	20 (61%)
Parent (very good/good at 4 hours)	20 (63%)	6 (19%)	p 0.0001	24 (73%)
Investigator (very good/good at 1 hour)	15 (47%)	8 (25%)	p < 0.0316	20 (61%)
Investigator (very good/good at Day 4)	22 (69%)	20 (63%)	-	27 (82%)

P values refer to the comparison between paracetamol and placebo.

### Efficacy evaluation over time of ketoprofen lysine salt

The efficacy of open-label ketoprofen-treated patients group, was similar to paracetamol, and definitely different from placebo (Figure 2, 3 and Table 2).

### Safety evaluations

Safety evaluations at 1, 4 hours after administration was rated good or very good by parents, investigators and children in more than 90% of the cases for both paracetamol and placebo. No serious adverse events occurred. Four adverse events were observed in 4 patients: bronchitis and rash in the ketoprofen lysine salt group, diarrhoea and cough in the placebo group; none of the event were related to the administered drugs or placebo.

### Blinding

No patients or physicians were unblinded to the paracetamol or placebo treatment.

## Discussion

In this randomised double blind trial children treated with paracetamol showed greater improvement in pain over time with respect to placebo-treated subjects, with similar effects observed in the open label ketoprofen-treated group.

In the past, the use of analgesics in the pediatric field was not fully adequate especially because parents were often under the misapprehension that analgesic drugs could have been harmful [12,13]. Pain is a part of life and effective analgesia in relation to the intensity of suffering should be provided either in the hospital setting, ambulatory care and home. The assessment and treatment of pain are meaningful parts of pediatric practice and analgesic drugs have been effectively used so far in neonates, infants and children [1,2]. However, the lack of adequate drug pediatric labeling and clinical trials in children called both the Food and Drug Administration (FDA) and the European Medicines Agency (EMA) for a legislative intervention with the aims to facilitate studies in children [14-16] and establish pediatric network [17-19]. This framework helped to facilitate the conduct this trial in the ambulatory care setting thanks to the Italian national wide net of family pediatrician.

In this double-blind, placebo-controlled clinical trial a 12 mg/Kg dosage of paracetamol in syrup was tested in children suffering of sore throat due to pharyngotonsillitis, and using as active control ketoprofen lysine salt administered in an open fashion. The after treatment analgesic effect was independently assessed by the investigator for 1 hour, and by child and parent for 4 hours using validated tools for the assessment of pain relief [6,20]. Both active drugs (paracetamol and ketoprofen lysine salt) were more effective than placebo in reducing sore throat, as reported by children and parents while no statistically significant differences between paracetamol and placebo were detected by investigators. This discrepancy may be related to the shorter period of the investigators' observation (1 hour only against the 4 hours of children and parents) and to the known discrepancy in physicians versus parents/children assessment [21-23]. Neverthless, when analgesic efficacy was evaluated using categorical scales, a statistically significant effect of paracetamol over placebo was detected by children, parents and investigators. The analgesic effect of ketoprofen was similar to that observed with paracetamol. Tolerability of both drug was very good with only four minor adverse events were reported, 2 in the ketoprofen group and 2 in the placebo group.

The analgesic efficacy of paracetamol in the treatment of pain in children is widely recognized, even if few consistent experiences are available in literature. Schachtel et al rated ibuprofen and acetaminophen (at 15 mg/kg) as significantly effective compared with placebo (p < 0.05) in children with acute sore throat under double-blind, placebo-controlled conditions [6]. Bertin et al found that ibuprofen but not paracetamol (at 10 mg/kg) was superior to placebo on day 2 for pain control in a double blind placebo-controlled trial in children with otitis media [24] or pharyngitis [25]. Hamalainen et al [26] found that ibuprofen was twice as likely as acetaminophen to abort migraine within 2 hours versus placebo in their double blind trial. The meta-analysis by Perrott et al [27] showed that in children, single doses of ibuprofen (4-10 mg/kg) and acetaminophen (7-15 mg/kg) have similar efficacy for relieving moderate to severe pain, and similar safety as analgesics or antipyretics. More recently, Clark et al in a trial with a partial blinded randomisation, found that ibuprofen was superior to paracetamol, or codein for acute pain relief in children with musculoskeletal trauma referred to a paediatric emergency department [13].

## Conclusions

In conclusion, this study confirm that a single oral dose of paracetamol or ketoprofen lysine salt are safe and effective analgesic treatments for children with sore throat in daily pediatric ambulatory care.

## Competing interests

ES, ADV, PD are employees of ACRAF Italy S.p.A.

The IRCCS G. Gaslini (where NR, ODCA and AM are full time employees)and the investigators (LC, RJ, FF, GP, MZ) received a research grant for the conduct of the study.

No grant has been provide for the paper writing.

## Authors' contributions

NR, ODCA and AM had a pivotal role in study design (including protocol and case report form development), data collection (including local monitoring but excluding enrollment of individual patients), data analysis, data interpretation through the critical revision of the company study report. The very preliminary draft of the paper was written internally by the company and deeply revised by NR, ODCA and AM. Company had the right to revise the version to be submitted but the final decision about paper submission relied entirely on NR, ODCA and AM.

LC, RJ, FF, GP, MZ, enrolled patients into the study and revised critically the paper version to be submitted.

All authors have read and agreed to its content, and that any experimental research that is reported in the manuscript has been performed with the approval of an appropriate ethics committee.
